# Emerging Novel GII.P16 Noroviruses Associated with Multiple Capsid Genotypes

**DOI:** 10.3390/v11060535

**Published:** 2019-06-08

**Authors:** Leslie Barclay, Jennifer L. Cannon, Mary E. Wikswo, Annie R. Phillips, Hannah Browne, Anna M. Montmayeur, Roman L. Tatusov, Rachel M. Burke, Aron J. Hall, Jan Vinjé

**Affiliations:** 1 Division of Viral Diseases, National Center for Immunization and Respiratory Diseases, Centers for Disease Control and Prevention, Atlanta, GA 30329, USA; flb8@cdc.gov (J.L.C.); ezq1@cdc.gov (M.E.W.); aphillips0619@gmail.com (A.R.P.); mqu6@cdc.gov (H.B.); xda8@cdc.gov (A.M.M.); yqy9@cdc.gov (R.L.T.); lxx8@cdc.gov (R.M.B.); esg3@cdc.gov (A.J.H.); ahx8@cdc.gov (J.V.); 2 National Foundation for the Centers for Disease Control and Prevention Inc., Atlanta, GA 30329, USA; 3 Oak Ridge Institute for Science and Education, Oak Ridge, TN 37830, USA; 4 Cherokee Nation Assurance, Arlington, VA 22202, USA

**Keywords:** Norovirus, recombinants, dual-typing, molecular epidemiology, clinical outcomes, non-structural proteins, immune antagonism, GII.4 Sydney, herd immunity, GII.P16

## Abstract

Noroviruses evolve by antigenic drift and recombination, which occurs most frequently at the junction between the non-structural and structural protein coding genomic regions. In 2015, a novel GII.P16-GII.4 Sydney recombinant strain emerged, replacing the predominance of GII.Pe-GII.4 Sydney among US outbreaks. Distinct from GII.P16 polymerases detected since 2010, this novel GII.P16 was subsequently detected among GII.1, GII.2, GII.3, GII.10 and GII.12 viruses, prompting an investigation on the unique characteristics of these viruses. Norovirus positive samples (*n* = 1807) were dual-typed, of which a subset (*n* = 124) was sequenced to yield near-complete genomes. CaliciNet and National Outbreak Reporting System (NORS) records were matched to link outbreak characteristics and case outcomes to molecular data and GenBank was mined for contextualization. Recombination with the novel GII.P16 polymerase extended GII.4 Sydney predominance and increased the number of GII.2 outbreaks in the US. Introduction of the novel GII.P16 noroviruses occurred without unique amino acid changes in VP1, more severe case outcomes, or differences in affected population. However, unique changes were found among NS1/2, NS4 and VP2 proteins, which have immune antagonistic functions, and the RdRp. Multiple polymerase-capsid combinations were detected among GII viruses including 11 involving GII.P16. Molecular surveillance of protein sequences from norovirus genomes can inform the functional importance of amino acid changes in emerging recombinant viruses and aid in vaccine and antiviral formulation.

## 1. Introduction

Noroviruses are a major cause of sporadic and epidemic acute gastrointestinal illness worldwide [1,2,3]. Outbreaks most commonly occur in semi-closed settings, e.g., hospitals, long-term care facilities, restaurants, and schools, and are typically transmitted by food or person-to-person [4]. Norovirus affects people of all ages, and most healthy individuals resolve symptoms within 48–72 h, whereas immunocompromised, very young, and elderly individuals have a higher likelihood of more protracted or severe outcomes [5,6]. 

Norovirus is a non-enveloped positive-sense single-stranded RNA virus of approximately 7.5 kb in length within the family *Caliciviridae.* The genome is organized into three open reading frames (ORFs). ORF1 encodes a large polyprotein that is cleaved into six nonstructural proteins (NS1/2, NS3, NS4, NS5, NS6, and NS7). ORF2 and ORF3 encode major (VP1) and minor (VP2) capsid proteins, respectively [6]. VP1 is divided into the shell (S) domain and protruding (P) domain, with the latter consisting of P1 and P2 regions, where P2 flanks P1 on either side. Based on VP1, noroviruses are classified into at least seven genogroups (GI to GVII), which can be further divided into at least 30 genotypes [6,7]. Of these, GI, GII, and GIV viruses cause disease in humans, and the majority of illnesses are associated with GI and GII infections. 

The P2 subdomain of VP1 contains potential neutralizing antibody epitopes and interacts with histo-blood group antigens (HBGAs), which are a diverse family of carbohydrates that serve as binding ligands for virus entry [8,9]. However, other parts of the norovirus genome may play a role in immune antagonism and affect viral replication and fitness. NS1/2 and NS4 aid in Golgi disassembly, accommodating replication complex formation and impairing host protein secretion [10]. The minor structural protein, VP2, has been shown to play a critical role in the production of infectious virus, providing structural support to the viral capsid and negative regulation of the RNA-dependent RNA polymerase (RdRp) [11,12,13]. Recently, VP2 of feline calicivirus (FCV) was shown to form a portal-like assembly which may serve as a channel for endosomal release of the viral genome [14]. 

Recombination events along with point mutations within the norovirus genome are well-documented forces that drive norovirus evolution and possibly herd immunity [10,15]. Norovirus recombination most frequently occurs between the junction of ORF1 and ORF2 [16,17,18]. Although less frequent, recombination has also been demonstrated within ORF1 [19], ORF2 [20], and between ORF2/ORF3 [18]. The ORF1/ORF2 junction region is an important point of recombination as nonstructural and structural proteins can be exchanged affecting pathogenesis, fitness, and immune antagonism [21,22,23].

Since the mid-1990s, GII.4 viruses have been the predominant circulating strains worldwide. Likely due to herd immunity, older variants are replaced by new GII.4 variants every 2–4 years [6,24]. GII.4 Sydney viruses emerged in 2012 associated with a GII.Pe polymerase (GII.Pe-GII.4 Sydney) but in 2015, a recombinant GII.4 Sydney harboring a novel GII.P16 polymerase (GII.P16-GII.4 Sydney) emerged [25]. This strain replaced the GII.Pe-GII.4 Sydney strain and continues to be the predominant strain in the US through the 2018–2019 season [26]. Since 2015, multiple other genotypes harboring a nearly identical GII.P16 polymerase have been identified including GII.1, GII.2, GII.3, GII.10, and GII.12 [25,26]. This novel GII.P16 polymerase is genetically distinct from those associated with the GII.2, GII.16, and GII.17 viruses detected since 1975 and those associated with GII.2, GII.3, and GII.13 viruses detected since 2010 that continue to circulate with a low frequency [25]. In this study, we attempt to find molecular signatures that could explain how viruses with the novel GII.P16 differ from those with other polymerase types and explore whether viruses with the novel GII.P16 manifest more severe disease outcomes. Continued surveillance of the changing epidemiology of noroviruses will better inform the formulation and targeting of candidate vaccines and antivirals. 

## 2. Materials and Methods

### 2.1. Data Sources for Norovirus Typing and Outbreak Information

Genotype, sequences, and limited outbreak data (i.e., outbreak date, outbreak state, outbreak identifiers) from outbreaks uploaded to CaliciNet [25,27] from September 2009–August 2018 and sporadic cases from Bangladesh [28,29], Guatemala [30], Nicaragua [31], and Peru [29] were downloaded on September 21, 2018. Norovirus sequences were downloaded from GenBank using NCBI taxonomy browser on October 12, 2018. Epidemiologic information (i.e., setting, transmission, demographics, outcomes) from the National Outbreak Reporting System (NORS) [32] matching CaliciNet outbreaks from September 2013–December 2017 were downloaded on February 6, 2019.

### 2.2. Norovirus RT-PCR

Stool specimens, collected following routine norovirus outbreak surveillance by CaliciNet laboratories, were tested by real-time reverse transcription-polymerase chain reaction (RT-qPCR) targeting the ORF1/ORF2 junction region on outbreak specimens, as previously described [25]. Positive samples were dual typed by conventional RT-PCR targeting of a 579 bp product for GI viruses or a 570 bp product for GII viruses that includes the 3’-end of the polymerase region and 5’-end of the major capsid gene followed by product purification and Sanger sequencing, as described previously [25]. 

### 2.3. Complete Genome Sequencing (NGS)

A selection of samples (*n* = 124) were analyzed by next generation sequencing (NGS) (Table S1). The CDC’s Internal Program for Research Determination deemed that this study is categorized as public health non-research and that human subject regulations did not apply. Briefly, viral RNA was extracted from clarified 10% *w/v* stool suspensions using the QIAmp Viral RNA mini Kit (Qiagen, Germantown, MD, USA), with supplementary filtration and nuclease treatments, as previously described [33]. Viral nucleic acids were amplified using a previously published sequence-independent, single-primer amplification (SISPA) protocol [34,35]. cDNA libraries were generated using an Illumina Nextera XT DNA Library Prep Kit (Illumina, San Diego, CA, USA) followed by sequencing on an Illumina MiSeq platform (Illumina, San Diego, CA, USA) [36,37]. Raw reads were preprocessed by adaptor and primer removal, host sequence subtraction, sequence deduplication, and quality filtering with a Phred score cutoff of 30 before de novo assembly was performed using SPAdes, version 3.7 [38], with multiple k-mer lengths. A recruitment mapping approach utilizing the internal algorithm in the Geneious, version 11.1.2, software package (Biomatters Inc., Newark, NJ, USA) resolved the final consensus sequence for each specimen. Near complete norovirus genome sequences were uploaded to GenBank with accession numbers KX354132, KX354134, KX354135, KX354140, MK629457, MK752933-MK752949, MK753007-MK753021, MK753028-MK753036, MK754442-MK754447, MK756033-MK756038, MK762558-MK762570, MK762621-MK762641, MK762745-MK762746, MK764013-MK764022, MK764039-MK764043, MK773571, MK773580-MK773588, and MK775028-MK775032 (Table S1).

### 2.4. Epidemiological Characteristics of GII.P16 Viruses

Norovirus outbreaks (September 2013–December 2017) caused by capsid types associated with GII.P16 were linked to NORS entries using unique outbreak identifiers, reporting state, and date of first illness onset (within 90 days) in both systems, as previously described [39]. Genotypic and epidemiologic data from linked outbreak entries were imported into R (https://www.r-project.org/) for statistical analysis comparing GII.P16 and non-GII.P16 outbreaks. Odds ratios were calculated for transmission, setting, gender, age, symptomology, and magnitude (number of ill cases) of outbreak. Rate ratios were calculated for outcomes. Significant differences were determined using a Χ^2^ test. *p*-values ≤0.01 were considered statistically significant, allowing more stringency due to the low number of matching (NORS-CaliciNet) outbreaks included in the study. 

### 2.5. Sequence Selection and Data Analysis

Sequences from outbreaks and sporadic cases of AGE with dual-typing (polymerase and capsid) information were compiled together with GenBank sequences (downloaded October 12, 2018) and typed using the Human Calicivirus Typing Tool (https://norovirus.ng.philab.cdc.gov/) which reports genotype information for both capsid and polymerase sequences [7]. A subset of GenBank sequences (Table S2) with near complete polymerase- and capsid-encoding regions (missing no more than 50 nt at the 5’ or 3’ ends) were further analyzed with the near complete genome sequences analyzed in our study (Table S1). Nucleotide sequences were aligned using MAFFT v7 software [40,41]. Recombination Detection Program software (RDP v4.97) was used to confirm there were no additional recombination breakpoints within ORF1 and ORF2/3 sequences. Time-scaled phylogenies were constructed using the Bayesian Markov chain Monte Carlo (MCMC) method in the BEAST software package v1.10.4 [42,43] using alignments constructed with the earliest GII.P16 sequences reported in GenBank (AB684676 and AY682551) and the 336 GII.P16 sequences identified in this study (Tables S1 and S2) were. The GTR + G + I substitution model was selected based on the best fit maximum likelihood model selection tool in MEGA v10.0.5. We used TempEst v1.5.1 to verify temporal association of phylogenies, chose an uncorrelated relaxed clock based on branching patterns indicative of different evolutionary rates and used a coalescent exponential growth tree prior. The MCMC was run on chain lengths of 100,000,000 steps with sampling every 10,000 steps. Data output were analyzed by Tracer v1.7.1 software (http://beast.community/tracer), confirming Effective Sample Size (ESS) values of ≥200 for acceptance. Maximum clade credibility (MCC) trees were created with 10% burn-in using the TreeAnnotator v1.10.4 and time-scaled trees were visualized using FigTree v1.4.3. 

To identify amino acid sites unique to viruses with the novel GII.P16 polymerase, nucleotide alignments were parsed into coding regions for structural and nonstructural proteins and translated using BioEdit v7.2.5 (http://www.mbio.ncsu.edu/BioEdit/bioedit.html). Proteins missing >16 amino acids at the 5’ or 3’ ends were excluded. Sequences were exported into MS Excel (Office 365) as tab-delimited alignments. Intra-group informative sites (locations where >10% sequences had an amino acid that differed from the dominant one of the groups) were first identified among viruses with the novel GII.P16 polymerase. Next, consensus sequences for viruses within this group were constructed for each viral protein by choosing the dominant amino acid (shared by >50% of sequences) at each site. Groups of protein sequences from viruses with different polymerase types (non-novel (extant) GII.P16, GII.Pe, GII.P4 New Orleans and GII.P2) were compared to the corresponding consensus sequences of novel GII.P16 viruses and inter-group informative sites (locations where >10% of sequences differed) were selected for further analysis. The relative frequency of amino acid occurrence (bits), grouped by amino acid chemistry (polar, neutral, basic, acidic, and hydrophobic) at informative sites were visualized using sequence logos (WebLogo v3; http://weblogo.threeplusone.com/) for each protein and each polymerase. Amino acid changes that were unique to viruses with the novel GII.P16 (and not found in other polymerase types) were also visualized alone using sequence logos.

## 3. Results

### 3.1. Emergence of A Novel GII.P16 Polymerase Lineage Detected in Outbreak Samples

**Figure 1:**
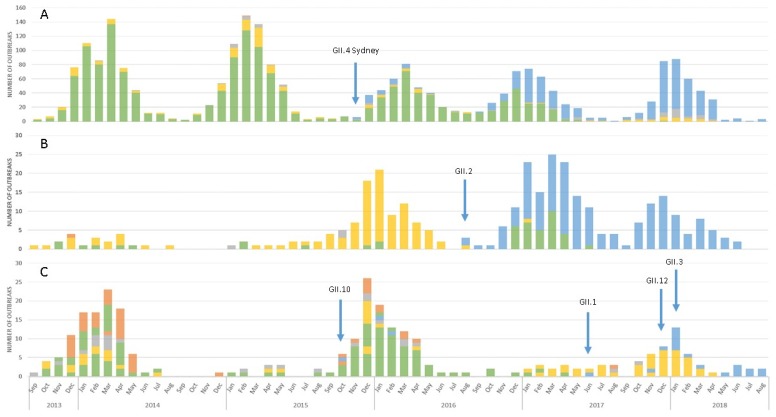
Outbreak distribution and emergence of a novel GII.P16 polymerase among (**A**) GII.4 Sydney (*n* = 2355), (**B**) GII.2 (*n* = 329) and (**C**) GII.1, GII.3, GII.10, GII.12, and GII.13* (*n* = 291). Noroviruses containing a novel GII.P16 polymerase corresponding to GII.4 Sydney, GII.2, GII.10, GII.1, GII.12, and GII.3 are shown in blue. Other polymerase types associated with GII.4 Sydney include GII.Pe (yellow), GII.P4 (grey), extant GII.P16 (orange), and unknown polymerase types (green). Other polymerase types associated with GII.2 include GII.P2 (yellow), GII.Pe (grey), extant GII.P16 (orange), and unknown polymerase types (green). GII.Pg-GII.1, GII.P12-GII.3, GII.Pc-GII.10, and GII.Pg-GII.12 viruses (yellow), GII.P21-GII.3, GII.Pg-GII.10, GII.Pq-GII.13 viruses (grey), GII.P16-GII.3 and GII.P16-GII.13 (extant) viruses (orange), and unknown polymerase types associated with GII.1, GII.3, and GII.13 capsids (green). The blue arrows indicate month and year of first report of novel GII.P16 polymerase associated with noted capsid. *GII.13 is only associated with extant GII.P16 and is included in the figure due to its association with GII.P16.

Of the 4,123 norovirus outbreaks submitted to CaliciNet between September 2013 and August 2018, polymerase and capsid sequences were available for 1808 outbreaks (43.9%). Viruses harboring the GII.P16 polymerase were detected in 43.1% (780/1808) of outbreaks. In 2013 and 2014, GII.P16 polymerases were associated primarily with GII.13 viruses but were also found among GII.2 and GII.3 viruses. We previously reported the emergence of a phylogenetically distinct (>5% nt difference in the polymerase-encoding region from GII.P16 polymerases detected prior to 2015) GII.P16 among GII.4 Sydney (2015) and GII.2 (2016) [25]. Our data showed that GII.P16-GII.4 Sydney (Figure 1A) and GII.P16-GII.2 (Figure 1B) strains continued to dominate in the 2016–2017 and 2017–2018 seasons, replacing the previously dominant GII.Pe-GII.4 Sydney and GII.P2-GII.2 strains, respectively. This novel GII.P16 was also detected among outbreaks involving GII.1 (*n* = 3), GII.3 (*n* = 1), GII.10 (*n* = 3), and GII.12 (*n* = 15) viruses (Figure 1C). 

**Figure 2:**
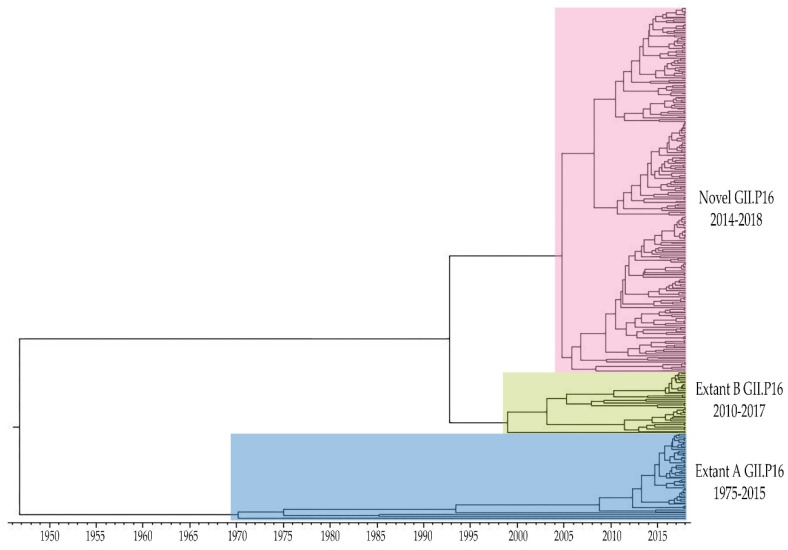
Time-scaled phylogenetic tree of complete GII.P16 polymerase nucleotide sequences constructed using the Bayesian MCMC method (BEAST v1.10.4) using the GTR + G + I substitution and uncorrelated relaxed clock model. Viruses in the Novel GII.P16 lineage were first detected in 2014 diverging from a common ancestor with Extant B GII.P16 viruses that were first reported in 2010. Viruses in the Extant A GII.P16 lineage were detected from 1975 to 2015. Figure includes all sequences from GenBank (Table S1) and CDC-generated (Table S2) included in this study.

Time-scaled phylogeny of GII.P16 polymerases showed distinct lineages of the GII.P16 polymerase which we distinguish as “novel”, “extant B” and “extant A” (Figure 2). Viruses in the novel GII.P16 lineage (GII.1, GII.2, GII.3, GII.4 Sydney, GII.10, and GII.12) have been detected since 2014 and were shown to diverge from a common ancestor with extant B strains. Within the extant B group (GII.2, GII.3, and GII.13), more recent strains diverged from a cluster of GII.2 and GII.3 strains detected in 2010–2012. Extant B and extant A strains diverged from a common ancestor, the oldest of which were found among GII.16 and GII.17 viruses. While extant B viruses continue to circulate in the US, they were less frequently detected than novel GII.P16 viruses. 

### 3.2. Polymerase-Capsid Combinations

**Figure 3:**
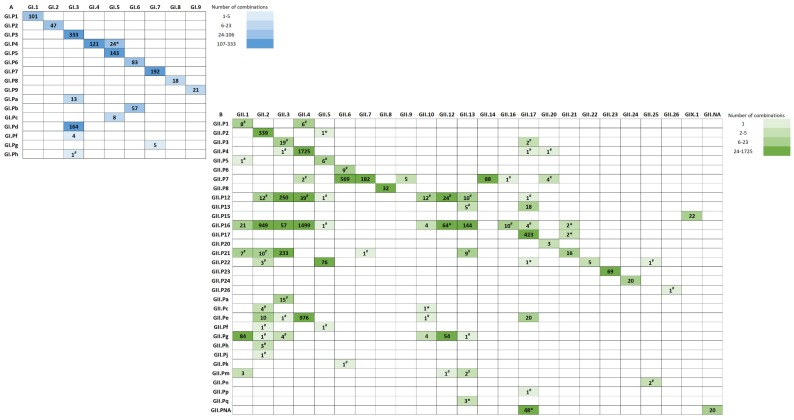
Norovirus polymerase and capsid dual type combinations of (**A**) Genogroup I (*n* = 1343) and (**B**) Genogroup II (*n* = 8294). Polymerase-capsid combinations denoted with an asterisk (*) were found in the CDC collection only. Polymerase-capsid combinations denoted with a hash (#) were found in GenBank only. NA: Not Assigned. Total polymerase breakdown for each type: GI.P1 (101), GI.P2 (47), GI.P3 (333), GI.P4 (145), GI.P5 (143), GI.P6 (83), GI.P7 (192), GI.P8 (19), GI.P9 (21), GI.Pa (13), GI.Pb (57), GI.Pc (8), GI.Pd (164), GI.Pf (4), GI.Pg (5), GI.Ph (1), GI.Pi (2), GII.P1 (14), GII.P2 (340), GII.P3 (21), GII.P4 (1728), GII.P5 (7), GII.P6 (9), GII.P7 (851), GII.P8 (32), GII.P12 (349), GII.P13 (23), GII.P15 (22), GII.P16 (2755), GII.P17 (425), GII.P20 (3), GII.P21 (276), GII.P22 (86), GII.P23 (69), GII.P24 (20), GII.P26 (1), GII.Pa (15), GII.Pc (5), GII.Pe (1008), GII.Pf (2), GII.Pg (148), GII.Ph (3), GII.Pj (1), GII.Pk (1), GII.Pm (6), GII.Pn (2), GII.Pp (1), GII.Pq (3). GII.P4 includes Den Haag (49), New Orleans (217), and 1462 sequences not typed to the variant level. Total capsid breakdown for each type: GI.1 (101), GI.2 (47), GI.3 (520), GI.4 (121), GI.5 (175), GI.6 (140), GI.7 (197), GI.8 (18), GI.9 (21), GII.1 (124), GII.2 (1333), GII.3 (580), GII.4 (4247), GII.5 (86), GII.6 (579), GII.7 (183), GII.8 (32), GII.9 (5), GII.10 (22), GII.12 (143), GII.13 (174), GII.14 (88), GII.16 (11), GII.17 (519), GII.20 (8), GII.21 (20), GII.22 (5), GII.23 (69), GII.24 (20), GII.25 (3), GII.26 (1), GIX.1 (22). GII.4 includes Den Haag (43), New Orleans (52), Sydney (2211), and nine typed outside our variant level threshold. Polymerase-capsid combinations that are not shown include GI.P8-GII.4 (1) was only found in GenBank and GI.P untypeable-GI.3 (5) and GI.Pi-GI.untypeable (2) which were found in strains sequenced in this study only. Sequences were downloaded from CaliciNet on September 21, 2018 and from GenBank on October 12, 2018. Sequence length used to type polymerase and capsid was at least 509 nt for GI and 497 nt for GII. The number of sequences available for each combination were calculated into quartiles separately for GI and GII. Each quartile was assigned a color transitioning from lighter to darker shades of blue for GI and green for GII.

Since multiple capsid types were associated with GII.P16 polymerases in our data set, we investigated whether recombination with multiple capsid types is unique to GII.P16 or a phenomenon shared by other polymerases using an expanded set of sequences. Among the available 1343 GI and 8294 GII strains with both polymerase and capsid sequences, 107 combinations were detected including 20 GI (Figure 3A) and 87 GII (Figure 3B). GI combinations had an almost 1:1 ratio of polymerase to capsid type, except GI.P4, GI.3, GI.5, GI.6, and GI.7. GII combinations did not hold the same ratio. More than one polymerase was detected for 63% (15/24) GII capsid types. While most GII polymerase types were associated with one capsid type, 9/32 (28%) were associated with multiple (>2) capsid types including GII.P16 (*n* = 11), P12 (*n* = 8), P7 (*n* = 7), P21 (*n* = 6), Pg (*n* = 6), P22 (*n* = 5), Pe (*n* = 5), P4 (*n* = 4), and Pm (*n* = 3).

### 3.3. Epidemiologic Characteristics and Outcomes for Novel GII.P16 Strains

Epidemiologic characteristics of outbreaks involving the novel GII.P16 viruses were compared to those with the same capsid type, but with different polymerases (including non-novel GII.P16s and other polymerase types), using NORS records that could be matched to CaliciNet outbreaks. Of the 3118 outbreaks meeting these criteria, 1828 produced matching records, and 598 outbreaks were available for further epidemiologic analysis. Excluded records included those not finalized in NORS (*n* = 195) and CaliciNet records with no polymerase data (*n* = 1035). We found no significant association between the novel GII.P16 polymerase and transmission routes, gender, age, or symptoms (Table 1 and Table 2). However, the novel GII.P16 polymerase was significantly more common among outbreaks in healthcare settings (OR 1.57, 99% CI: 1.13–2.17) (Table 1), and was associated with a significantly lower rate of emergency department visits (RR 0.69, 99% CI: 0.52–0.91) (Table 3). 

### 3.4. Unique Markers of Viruses with the Novel GII.P16 Polymerase

**Figure 4:**
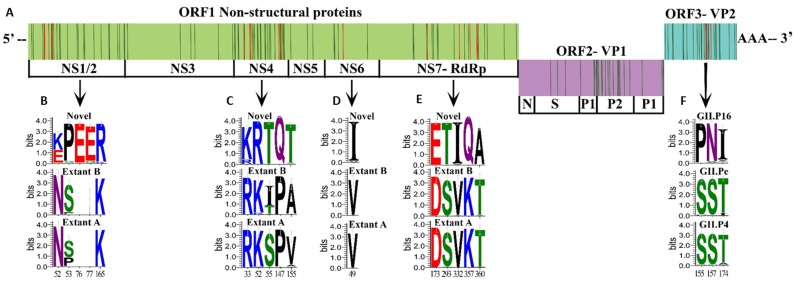
Informative and unique amino acid sequences detected among viruses with the novel GII.P16 polymerase. (**A**) Genomic organization of norovirus with informative sites (locations where >10% of sequences with a different polymerase type differed from those with a novel GII.P16 polymerase that emerged in 2015) are indicated with a vertical line (red, indicates changes at informative sites resulting in an amino acid that was unique to novel GII.P16 strains; green, indicates informative sites that are not unique to novel GII.P16 strains). (**B**–**E**) Sequence logos indicating the relative frequency of amino acid occurrence (bits) for informative sites unique to novel GII.P16 viruses within non-structural proteins when compared to Extant B and Extant A GII.P16 polymerases. Unique informative sites shown for (**B**) NS1/2, (**C**) NS4, (**D**) NS6, (**E**) NS7. (**F**) Sequence logos of informative sites unique to novel GII.P16-GII.4 Sydney viruses within VP2 when compared to GII.4 Sydney viruses with GII.Pe and GII.P4 New Orleans polymerases. Bottom numbers indicate amino acid position within each protein.

Because GII.4 Sydney and GII.2 strains that harbor the novel GII.P16 polymerase became predominant outbreak strains in the US, during the 2015/2016 and 2016/2017 season, respectively, replacing those with other polymerases, we looked for amino acid changes unique to novel GII.P16 viruses that may result in advantageous structural or functional changes (Figure 4A). For NS1/2, 26 informative sites were found (Figure S1F), four of which were unique to the novel GII.P16 group (Figure 4B). Amino acid chemistry was altered at N52K/E, with 56% strains in the novel GII.P16 group with a basic K52 and 42% with an acidic 52E (a G or T was present for 3 strains) (Figure 4B). In addition, unique to the novel GII.P16 group was a two amino acid insertion at site 76 and 77 (Figure 4B), resulting in a densely acidic region composed of five adjacent glutamic acid residues (76–80) next to a proline rich motif (69–73). We found 28 informative sites (Figure S1D) within p22 (NS4), five of which were unique to the novel GII.P16 group and two resulted in a change in amino acid chemistry (P147Q and A/V155T) (Figure 4C). There were six informative sites in the NTPase (NS3) (Figure S1E), none of them unique to the novel GII.P16 group. In the protease (NS6) region five informative sites were identified (Figure S1B), one of which was unique to the novel GII.P16 group (Figure 4D). Three informative sites were identified for VPg (NS5) (Figure S1C), none of which were unique to the novel GII.P16 group. Within the polymerase protein (NS7), we detected 20 informative sites (Figure S1A), five of which were unique to the novel GII.P16 when compared to extant strains (Figure 4E) and two of which resulted in a change in amino acid chemistry (K357Q and T360A). 

**Figure 5:**
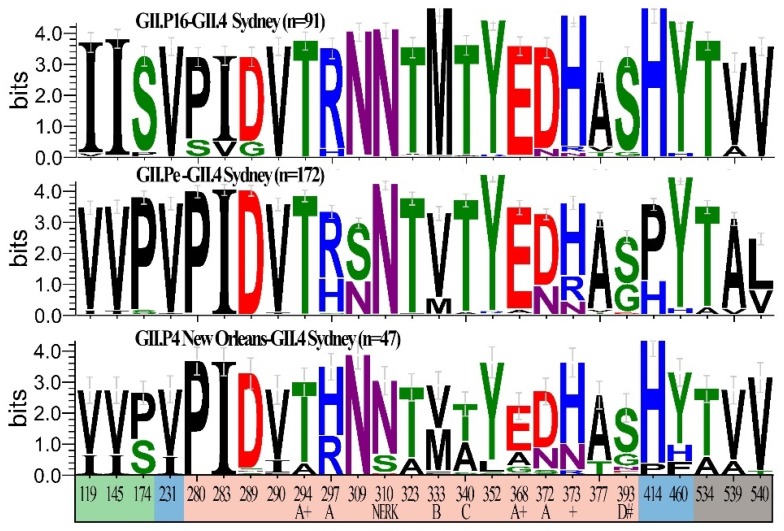
Informative amino acid sequences within the VP1 protein of GII.4 Sydney viruses with and without the novel GII.P16 polymerase. Sequence logos indicating the relative frequency of amino acid occurrence (bits) for locations where >10% of sequences with a different polymerase type differed from those with a novel GII.P16 polymerase for GII.4 Sydney strains with a novel GII.P16 (*n* = 91 sequences), GII.Pe (*n* = 172 sequences) or GII.P4 New Orleans (*n* = 47 sequences) polymerase. Bottom numbers indicate amino acid position within VP1 and colors indicate Shell (green), Protruding one (blue), Protruding 2 (peach) and C-terminal (grey) domains. Sites involved in neutralizing antibody recognition (epitopes A, B, C and D), the NERK motif, and histo-blood group antigen binding (#), as well as those under positive selection (+) are indicated.

For GII.4 Sydney strains, there were 26 informative sites within VP1 (Figure 5). Although some of these informative sites coincided with important regions for antibody recognition and HBGA binding (Figure 5), none of those sites were unique to strains with the novel GII.P16 polymerase. There were also 26 informative sites found within VP2 of GII.4 Sydney strains (Figure S2). Of these, three were unique to those with a novel GII.P16 (Figure 4F), each of which resulted in a change in amino acid chemistry (S155P, S157N, and T174I). GII.2 strains were associated with extant and novel polymerases in addition to GII.P2. We found 20 informative sites within VP1 (Figure S3A), none of which were unique to the novel GII.P16 group. However, within VP2, there were 24 informative sites (Figure S3B) found, one of which was unique to novel GII.P16 group (G109N). 

## 4. Discussion

Recombination among noroviruses most frequently occurs in the ORF1-ORF2 junction region. Between 2015–2018, a novel GII.P16 polymerase was detected among GII.1, GII.2, GII.3, GII.4 Sydney, GII.10, and GII.12 viruses. GII.4 Sydney and GII.2 viruses with this polymerase predominated and replaced GII.Pe-GII.4 and GII.P2-GII.2 viruses that circulated in previous years. Interestingly, replacement of ORF1 sequences, including the polymerase region, occurred without unique amino acid changes in VP1 of these viruses. In contrast, Lindesmith et al., [44] found escape mutations within epitopes A, B, and D and the NERK motif that occurred with the introduction of the novel GII.P16-GII.4 Sydney using in vitro studies [44]. However, the GII.Pe- and GII.P16- GII.4 Sydney strains that they used to generate the VLPs have unique amino acid changes not shared by the majority of GII.Pe-GII.4 Sydney strains published to date. In our study, we included a much larger number of GII.4 Sydney strains in our analysis and observed fluctuation and recycling of VP1 amino acids within and among strains with different polymerase types, such that no motifs could uniquely be recognized among GII.P16-GII.4 Sydney viruses. Studies mapping the location of neutralizing antibody epitopes of GII.2 viruses are lacking, but it is not surprising that GII.2 VP1 amino acids unique to viruses with the novel GII.P16 were not found since GII.2 capsids lack the plasticity of GII.4 viruses [45].

Emergence of new norovirus strains with the novel GII.P16 polymerase occurred in the US without obvious changes in route of transmission, virulence or affected population. While GII.P16-GII.2 viruses caused increased numbers of cases among children in Germany [46] and Taiwan [47] and young adults in China [48], such an association could not be determined in our study, as outbreaks captured in CaliciNet more commonly involve adults and elderly persons. Healthcare settings were significantly associated with novel GII.P16 viruses in our data set, but an older age (≥75) was not. Using a larger set of matched NORS-CaliciNet data (but without available polymerase typing data), our group previously reported a significant association among GII.4 Sydney viruses in healthcare settings and age (≥75) [39]. In the current study when GII.4 Sydney or GII.2 strains were examined individually by setting (healthcare vs non-healthcare) and polymerase type (novel GII.P16 vs other polymerase types), no association was found (data not shown), indicating healthcare is a frequent setting for GII.4 Sydney viruses regardless of which polymerase they harbor. Increased hospitalization and death rates are typically attributed to GII.4 strains [39]. In our study, not only did we find no significant increases in outcomes, we found emergency department visit rates were significantly lower among outbreaks associated with the novel GII.P16 polymerase as opposed to non-GII.P16 outbreaks. However, previous studies have found emergency department visits to be commonly associated with norovirus infections [49,50]. Due to the possibility our results are confounded by setting and/or age, in the future, with more data points, we suggest performing a multivariate analysis on the outcomes stratified by setting and age. It is possible that patients already undergoing medical care are admitted directly as hospitalized inpatients, resulting in lower rates of emergency department visits. From our study, introduction of GII.P16 polymerase appears not to influence virulence; contrarily, recombination with the novel GII.P16 may have introduced mutations enhancing viral fitness rather than those that would exploit immunological naivety in the population.

Several GII polymerases were associated with multiple capsid types, especially those associated with GII.P16 (*n* = 11), GII.P12 (*n* = 8), GII.P7 (*n* = 7), GII.P21 (*n* = 6) and GII.Pg (*n* = 6). Interestingly, a recent analysis of the evolutionary rates of 25 polymerase types showed the highest rates among GII.P16 and GII.P12 viruses and suggested that the RdRp region evolves rapidly, similar to VP1 [51]. Such evolution could increase its likelihood for recombination. Our findings on the quantity of P-C combinations observed are similar to those reported in a recent review [15], although, some important differences were found due primarily to the methodologies used. For example, we detected four, rather than 10 capsid types associated with a GII.P4 polymerase. The discrepant sequences either did not type as GII.P4 using the NoroNet tool (https://www.rivm.nl/mpf/typingtool/norovirus), even though originally published as GII.P4 [15], or were not captured in our analysis due to the algorithms used for sample selection necessitating sequences match (region B: ≥87% for GI or ≥93% for GII; region C: ≥90% identity) with the tool’s reference sequences and they include both polymerase (region B: ~ 170 nt) and capsid (region C: ~ 250–260 nt) sequences. We also report a greater number of capsids associated with GII.P16 in our study, but since our sequence data was extracted more recently, coinciding with the increased accessibility of NGS technologies for whole genome sequencing, it is difficult to make unbiased comparisons of polymerase recombination frequency using GenBank data.

Multiple amino acid changes unique to GII.P16 viruses were found among viral proteins VP2, NS1/2, and NS4 which have immune antagonistic functions [10]. VP2 provides structural stability to capsids, associates with viral RNA, and negatively regulates RdRp activity [11,12,13]. Whether or not human norovirus VP2 proteins form the pore-like structures of FCV VP2 proteins facilitating release of viral RNA [14] is yet to be determined. NS1/2 and NS4 promote Golgi disassembly, interrupting cellular secretory activity and hindering trafficking of immune defense molecules such as cytokines, major histocompatibility complex (MHC) and costimulatory factors. All unique informative sites of the NS1/2 proteins of novel GII.P16 viruses were located in the disordered *n*-terminal portion of NS1. The two amino acid insertion in NS1 was located in a site sharing similarity with the Vesicle-associated membrane protein-associated protein A (VAPA)-binding region of murine norovirus (MNV) [52]; human norovirus NS1 also associates with intercellular VAPA, a critical regulator of soluble NSF attachment protein receptor (SNARE)-mediated vesicle fusion [53]. The unique NS4 amino acid changes detected in viruses with the novel GII.P16 were not located in the regions known to be membrane associated or in the MERES site, which is necessary for blocking endoplasmic reticulum (ER) to Golgi transport of coat protein complex II (COPII)-coated vesicles [54]. Interestingly, three of the unique changes among novel GII.P16 viruses occurred within the 5’ end of NS4, which was reported to be immunogenic in GI.1 viruses [55], opening the possibility that NS4 has an extracellular role in immune antagonism. 

In addition, amino acid changes were detected in the palm region between motifs C and D which is outside of the known catalytic motifs of RdRp. This region has an unclear impact on polymerase fidelity and recombination potential, although a palm motif of picornavirus RdRp is responsible for fine-tuning replication fidelity by closing the active site [56]. Amino acid 291 in the finger region between motifs A and B is important for controlling norovirus RdRp replication rate in vitro as GII.4 strains detected from 2001–2006 containing a T291 or V291 demonstrated higher radionucleotide incorporation rates when compared to GII.4 1995 (Lordsdale) strains which contained a K291 [21]. This mutation increased replication rate but did not affect rates of mutation. All GII.P16 virus clades detected herein contained predominantly the positively charged K291 as opposed to the polar, uncharged Q291 of GII.Pe-GII.4 Sydney and the T291 of GII.P4 New Orleans-GII.4 Sydney and GII.P2-GII.2 strains (data not shown), suggesting that introduction of the novel GII.P16 could have reduced the elongation rates of these viruses. GII.1, GII.10 and GII.12 viruses with the novel GII.P16 were not detected in epidemic proportions, but perhaps this is not surprising since these genotypes are uncommonly detected in CaliciNet, suggesting these capsid genotypes are less successful than GII.4 viruses regardless of polymerase association. In vitro studies showed that even though non-GII.4 virus (GII.3 and GII.7) replication rates in the study were similar to those of GII.4 viruses, they had low rates of mutation resulting in decreased epidemiological fitness [21]. 

Our attempts to identify amino acid signatures unique to viruses with the novel GII.P16 is challenging, since the changes were not concentrated in the protruding domain of VP1, but rather in non-structural proteins (NS1/2 and NS4) and VP2 which have roles in replication complex formation and immune antagonism but for which less information on the structure and location of epitopes necessary for their antagonistic function is known. In addition, we identify finger region changes in the RdRp that control rates of virus elongation, but less is known about the palm regions changes we identify that may relate to replication fidelity. It is tempting to speculate that the changes we identified are the driver for GII.4 Sydney and GII.2 virus replacement by strains with the novel GII.P16, but in vitro studies are needed to support this hypothesis. Our work can guide mutant construction and protein expression studies; amino acids from extant and novel GII.P16 polymerases can be interchanged and their impact on viral replication and clearance mechanisms studied in cell culture. Furthermore, the recently described human enteroid [57] and B-cell culture models [58] are also important tools to further study the virulence, fitness, and immune evasion and antagonism of new recombinant viruses such as those with the novel GII.P16.

## 5. Conclusions

We report the emergence of a novel GII.P16 polymerase that is associated with multiple capsid types. Introduction of the novel polymerase was associated with predominance of GII.P16-GII.4 Sydney and GII.P16-GII.2 viruses replacing the previous GII.Pe-GII.4 Sydney and GII.P2-GII.2 strains, which occurred without unique amino acid changes in the VP1 epitopes known to be important for immune escape and HBGA binding. We integrated norovirus typing data from CaliciNet and epidemiologic data from NORS and found no increases in hospitalization or death rates among viruses harboring a novel GII.P16 polymerase, suggesting viral fitness was enhanced but virulence was not. Focusing on different genotypes associated with the novel GII.P16 polymerases, we added a significant number (*n* = 124; Table S1) of near complete or complete genome sequences to GenBank, which helped us characterize unique amino acid changes across the genome. We selected sequences from multiple geographically distinct outbreaks and sporadic cases occurring in the US within a broad timespan (2013–2018) and identified nine additional polymerase capsid combinations that were not available in GenBank at the time of our query. Unique changes were detected among viral proteins having roles in immune antagonism (NS1/2, NS4 and VP2) as well as in the RdRp region which could impact replication rate and fidelity. Continued norovirus surveillance using routine dual typing of strains will assist in determining the functional importance of unique amino acids found among viruses emerging with the novel GII.P16 polymerase. These data can help guide the formulation, targeting, and anticipated impacts of future norovirus vaccines and antivirals. 

## Figures and Tables

**Table 1:** Number of norovirus outbreaks by polymerase type, transmission, and outbreak setting *.

**Transmission**	**Other Polymerases** **Total (%)**	**Novel GII.P16** **Total (%)**	**OR (99% CI)**	**Total No.**
Foodborne	74 (54.4)	62 (45.6)	1.00	136
Person-to-person	184 (48.8)	193 (51.2)	1.25 (0.75–2.11)	377
Other	47 (55.3)	38 (44.7)	0.97 (0.47–1.98)	85
**Setting**	**Other Polymerases** **Total (%)**	**Novel GII.P16** **Total (%)**	**OR (99% CI)**	**Total No.**
Non-Healthcare	154 (56.6)	118 (43.4)	1.00	272
Healthcare	144 (45.4)	173 (54.6)	1.57 (1.02–2.41) **	317

* Other Polymerases include GII.P2, GII.P12, extant GII.P16, GII.P17, GII.P21, GII.Pe, GII.Pg, and GII.Pq; OR: Odds ratio; Other transmission routes include environmental (*n* = 3), indeterminate/other/unknown (*n* = 79), and waterborne (*n* = 3). Non-Healthcare settings include Banquet Facility (*n* = 9), Beach – Public (*n* = 1), Camp (*n* = 5), Caterer (*n* = 25), Child day care (*n* = 23), Event space (*n* = 14), Office/indoor workplace (*n* = 2), Other (*n* = 7), Prison/jail (*n* = 5), Private home/residence (*n* = 18), Religious facility (*n* = 2), Restaurant (*n* = 104), School/college/university (*n* = 57). Healthcare settings include Hospital (*n* = 20), Long-term care/nursing home/assisted living facility (*n* = 294), and Other healthcare facility (*n* = 3). ** Statistically significant (*p*-value: 0.007).

**Table 2:** Number of norovirus outbreaks by polymerase type, gender, age, symptoms, and magnitude of outbreak *.

Characteristics of Outbreaks		Other Polymerases Total (%)	Novel GII.P16 Total (%)	OR (99% CI)	Total No.
**Gender**	<50% Female	88 (55.0)	72 (45.0)	1.00	160
	≥50% Female	187 (54.5)	156 (45.5)	1.02 (0.62–1.68)	343
**Age, y**	<50% 75 or older	181 (58.6)	128 (41.4)	1.00	309
	≥50% 75 or older	68 (52.3)	62 (47.7)	1.29 (0.75–2.22)	130
**Symptoms**					
Abdominal cramps	<50% cases	153 (54.1)	130 (45.9)	1.00	283
	≥50% cases	63 (55.8)	50 (44.2)	0.93 (0.52–1.66)	113
Fever	<50% cases	211 (52.2)	193 (47.8)	1.00	404
	≥50% cases	24 (66.7)	12 (33.3)	0.55 (0.20–1.39)	36
Vomiting	<50% cases	130 (51.8)	121 (48.2)	1.00	251
	≥50% cases	155 (56.6)	119 (43.4)	0.83 (0.52–1.30)	274
Diarrhea	<50% cases	87 (43.4)	76 (46.6)	1.00	163
	≥50% cases	195 (53.6)	169 (46.4)	0.99 (0.61–1.62)	364
**Magnitude**	0–23 cases	165 (55.2)	134 (44.8)	1.00	299
	24–313 cases	140 (46.8)	159 (53.2)	1.40 (0.92–2.14)	299

* Other Polymerases include GII.P2, GII.P12, extant GII.P16, GII.P17, GII.P21, GII.Pe, GII.Pg, and GII.Pq; OR: Odds ratio; Magnitude is characterized based on estimated primary ill by outbreak and cut off was defined by the median.

**Table 3:** Case outcome by polymerase type *.

Outcome	Other Polymerases			Novel GII.P16			RR (99% CI)
	Case Counts	Total No.	Rate per 10,000 Cases	Case Counts	Total No.	Rate per 10,000 Cases	
Outpatient Visit	322	5086	633.11	282	4922	572.94	0.90 (0.73–1.11)
Emergency Department Visit	194	5428	357.41	156	6260	249.20	0.69 (0.52–0.91) **
Hospitalized	155	7848	197.50	179	8415	212.72	1.08 (0.81–1.43)
Death	17	8447	20.13	18	8718	20.65	1.03 (0.42–2.51)

* Other Polymerases include GII.P2, GII.P12, extant GII.P16, GII.P17, GII.P21, GII.Pe, GII.Pg, and GII.Pq; RR: Rate ratio. ** Statistically significant (*p*-value: 0.0006).

## Supplementary Materials

The following are available online at https://www.mdpi.com/1999-4915/11/6/535/s1, Figure S1: Informative amino acid sequences within non-structural proteins of viruses with GII.P16 polymerases, Figure S2: Informative amino acid sequences within the VP2 protein of GII.4 Sydney viruses with and without the novel GII.P16 polymerase, Figure S3: Informative amino acid sequences within the structural proteins of GII.2 viruses with and without the novel GII.P16 polymerase, Table S1: Sequences generated with this study with typing information for near complete RdRp and VP1 coding regions, Table S2: Accession numbers, dual-typing information and GII.P16 subtypes for sequences with near complete RdRp and VP1 coding regions obtained from GenBank in this study.
