# Supplementary material for: Emerging Novel GII.P16 Noroviruses Associated with Multiple Capsid Genotypes
Source: Viruses. 2019 Jun 8;11(6):535. doi: 10.3390/v11060535 (PMC6631344; doi:10.3390/v11060535)
Supplement: Supplementary file 1 [file viruses-11-00535-s001.zip › Supplemental Table 1_Submission.docx]

**Supplemental Table 1.** Norovirus sequences (n=124) generated with this study with GenBank accession numbers, dual-typing information, and GII.P16 subtypes with near complete or complete VP1 (C-type) and RdRp (P-type) coding regions*

| **Title** | **GenBank accession** | **C-type#** | **P-type** | **GII.P16 subtype** |
| --- | --- | --- | --- | --- |
| CS-4809-2014 | MK762558 | GII.4 | GII.Pe | NA |
| CS-4963-2014 | MK762559 | GII.13 | GII.P16 | Extant B |
| CS-4964-2014 | MK762560 | GII.13 | GII.P16 | Extant B |
| Pierce-7119-2014 | KX354132 | GII.4 | GII.P4 | NA |
| Racine-7121-2014 | KX354134 | GII.4 | GII.P4 | NA |
| Waushara-7123-2015 | KX354135 | GII.4 | GII.P4 | NA |
| Pima-7351-2014 | MK753028 | GII.4 | GII.P4 | NA |
| CS-3201-2018 | MK762561 | GII.4 | GII.P4 | NA |
| Alameda-0066-2016 | MK756033 | GII.4 | GII.P16 | Novel |
| Alameda-0215-2016 | MK756034 | GII.4 | GII.P16 | Novel |
| Alameda-0236-2016 | MK756035 | GII.4 | GII.P16 | Novel |
| Alameda-0246-2016 | MK756036 | GII.4 | GII.P16 | Novel |
| Alameda-0259-2016 | MK756037 | GII.4 | GII.P16 | Novel |
| Alameda-0296-2016 | MK756038 | GII.4 | GII.P16 | Novel |
| CS-6257-2016 | MK762562 | GII.4 | GII.P16 | Novel |
| CS-6258-2016 | MK762563 | GII.4 | GII.P16 | Novel |
| CS-6259-2016 | MK762564 | GII.4 | GII.P16 | Novel |
| Stafford-6531-2016 | MK764018 | GII.4 | GII.P16 | Novel |
| San Diego-8911-2015 | MK753029 | GII.4 | GII.P16 | Novel |
| Wayne-8949-2016 | MK764013 | GII.4 | GII.P16 | Novel |
| Lane-0787-2015 | KX354140 | GII.4 | GII.P16 | Novel |
| Baltimore City-0832-2015 | MK754442 | GII.4 | GII.P16 | Novel |
| CS-0852-2016 | MK762565 | GII.13 | GII.P16 | Extant B |
| Marin-1324-2015 | MK753030 | GII.4 | GII.P16 | Novel |
| LaPorte-1329-2015 | MK762637 | GII.4 | GII.P16 | Novel |
| CS-4240-2016 | MK762566 | GII.4 | GII.P16 | Novel |
| CS-4242-2016 | MK762567 | GII.4 | GII.P16 | Novel |
| Summit-4812-2014 | MK775028 | GII.2 | GII.P2 | NA |
| Auglaize-4825-2016 | MK629457 | GII.4 | GII.P16 | Novel |
| CS-7680-2016 | MK762568 | GII.4 | GII.P16 | Novel |
| Dane-7952-2015 | MK752945 | GII.2 | GII.P2 | NA |
| Marathon-7989-2016 | MK752942 | GII.4 | GII.P16 | Novel |
| La Crosse-8016-2016 | MK752943 | GII.4 | GII.P16 | Novel |
| Washington-8070-2016 | MK753007 | GII.2 | GII.P2 | NA |
| Arlington-9929-2015 | MK764019 | GII.4 | GII.Pe | NA |
| Yolo-1756-2016 | MK753031 | GII.2 | GII.P2 | NA |
| Mecklenburg-1799-2016 | MK773580 | GII.2 | GII.P16 | Novel |
| Mecklenburg-1800-2016 | MK773581 | GII.2 | GII.P16 | Novel |
| Mecklenburg-1802-2016 | MK773582 | GII.2 | GII.P16 | Novel |
| Mecklenburg-1803-2016 | MK773583 | GII.2 | GII.P16 | Novel |
| Pittsylvania-1828-2014 | MK764020 | GII.3 | GII.P16 | Extant B |
| Sumner-2104-2014 | MK762640 | GII.3 | GII.P16 | Extant B |
| Davidson-2125-2016 | MK762641 | GII.2 | GII.P16 | Novel |
| Richland-2194-2016 | MK775029 | GII.2 | GII.P2 | NA |
| Winnebago-3105-2014 | MK752947 | GII.13 | GII.P16 | Extant B |
| Ozaukee-3285-2015 | MK752944 | GII.2 | GII.P2 | NA |
| Brown-3305-2015 | MK752946 | GII.13 | GII.P16 | Extant B |
| Multi-County-3505-2015 | MK762745 | GII.13 | GII.P16 | Extant B |
| Allegan-3652-2015 | MK764014 | GII.13 | GII.P16 | Extant B |
| Jackson-5326-2017 | MK762621 | GII.4 | GII.P16 | Novel |
| Multnomah-5650-2013 | MK753008 | GII.13 | GII.P16 | Extant B |
| Polk-5652-2013 | MK753009 | GII.13 | GII.P16 | Extant B |
| Marion-5661-2014 | MK753010 | GII.13 | GII.P16 | Extant B |
| Yamhill-5691-2015 | MK753011 | GII.2 | GII.P2 | NA |
| Douglas-5700-2016 | MK753012 | GII.2 | GII.P2 | NA |
| Clackamas-5703-2016 | MK753013 | GII.2 | GII.P2 | NA |
| Marion-5705-2016 | MK753014 | GII.2 | GII.P2 | NA |
| Lane-5706-2016 | MK753015 | GII.2 | GII.P2 | NA |
| Washington-6402-2014 | MK754443 | GII.13 | GII.P16 | Extant B |
| Jackson-6997-2017 | MK762622 | GII.4 | GII.P4 | NA |
| Jackson-7001-2017 | MK762623 | GII.4 | GII.P4 | NA |
| CS-7611-2017 | MK762569 | GII.4 | GII.P16 | Novel |
| CS-7612-2017 | MK762570 | GII.4 | GII.P16 | Novel |
| Jackson-3426-2017 | MK762624 | GII.4 | GII.P16 | Novel |
| Jackson-4513-2017 | MK762625 | GII.2 | GII.P16 | Novel |
| Natrona-0753-2017 | MK762632 | GII.4 | GII.P16 | Novel |
| Cerro Gordo-0770-2017 | MK752937 | GII.4 | GII.P16 | Novel |
| St. Croix-0775-2017 | MK752936 | GII.4 | GII.P16 | Novel |
| Brown-0777-2017 | MK752938 | GII.4 | GII.P16 | Novel |
| Pierce-0784-2017 | MK752939 | GII.2 | GII.P16 | Novel |
| Black Hawk-0791-2017 | MK752941 | GII.2 | GII.P16 | Novel |
| Polk-0797-2018 | MK752935 | GII.2 | GII.P16 | Novel |
| Linn-0842-2017 | MK753016 | GII.2 | GII.P16 | Novel |
| Lane-0848-2017 | MK753017 | GII.4 | GII.P16 | Novel |
| Clackamas-0850-2017 | MK753018 | GII.4 | GII.P16 | Novel |
| Ingham-0873-2017 | MK764015 | GII.4 | GII.P16 | Novel |
| Bay-0889-2018 | MK764016 | GII.4 | GII.P16 | Novel |
| Wood-0949-2018 | MK775032 | GII.4 | GII.P16 | Novel |
| Lee-1219-2017 | MK762629 | GII.4 | GII.P16 | Novel |
| San Joaquin-1241-2016 | MK753032 | GII.4 | GII.P16 | Novel |
| Cambria-1360-2016 | MK764039 | GII.2 | GII.P2 | NA |
| Cambria-1361-2016 | MK764040 | GII.2 | GII.P2 | NA |
| Cambria-1373-2018 | MK764041 | GII.12 | GII.P16 | Novel |
| Loudoun-1821-2017 | MK764021 | GII.4 | GII.P16 | Novel |
| Hanover-1833-2018 | MK764022 | GII.2 | GII.P16 | Novel |
| Baltimore-1834-2017 | MK754444 | GII.4 | GII.P16 | Novel |
| Somerset-1837-2017 | MK754445 | GII.12 | GII.P16 | Novel |
| Washington-1840-2018 | MK754446 | GII.4 | GII.P16 | Novel |
| Baltimore-1846-2018 | MK754447 | GII.12 | GII.P16 | Novel |
| New York-3591-2017 | MK762633 | GII.2 | GII.P16 | Novel |
| Grant-3893-2017 | MK752934 | GII.4 | GII.P16 | Novel |
| Grant-3894-2017 | MK752933 | GII.4 | GII.P16 | Novel |
| Miami-8721-2017 | MK762638 | GII.4 | GII.P16 | Novel |
| Porter-8724-2017 | MK762639 | GII.4 | GII.P16 | Novel |
| Los Angeles-8728-2017 | MK762635 | GII.4 | GII.P16 | Novel |
| Los Angeles-8734-2017 | MK762636 | GII.4 | GII.P16 | Novel |
| Olmsted-0757-2016 | MK773584 | GII.4 | GII.P16 | Novel |
| Olmsted-0760-2016 | MK773585 | GII.4 | GII.P16 | Novel |
| Ramsey-0768-2017 | MK773586 | GII.4 | GII.P16 | Novel |
| Hennepin-0772-2017 | MK773587 | GII.2 | GII.P16 | Novel |
| Unknown-1762-2016 | MK753035 | GII.2 | GII.P16 | Novel |
| Maricopa-1766-2016 | MK753033 | GII.1 | GII.P16 | Novel |
| Salt Lake-1249-2017 | MK753034 | GII.1 | GII.P16 | Novel |
| San Diego-1267-2017 | MK753036 | GII.12 | GII.P16 | Novel |
| Kenai Peninsula-1896-2014 | MK762626 | GII.2 | GII.P16 | Novel |
| Douglas-0900-2017 | MK762627 | GII.12 | GII.P16 | Novel |
| Ada-0910-2018 | MK762628 | GII.2 | GII.P2 | NA |
| Walworth-5203-2013 | MK752948 | GII.4 | GII.Pe | NA |
| Hood River-5186-2013 | MK753019 | GII.4 | GII.Pe | NA |
| Broward-6939-2015 | MK762630 | GII.4 | GII.Pe | NA |
| Hennepin-0774-2018 | MK773588 | GII.3 | GII.P16 | Novel |
| Rock-3065-2013 | MK752949 | GII.2 | GII.P16 | Extant A |
| Hood River-5673-2014 | MK753020 | GII.13 | GII.P16 | Extant B |
| Hardin-5832-2016 | MK762746 | GII.4 | GII.P16 | Novel |
| La Crosse-0781-2017 | MK752940 | GII.2 | GII.P16 | Novel |
| Multi-County-0838-2016 | MK753021 | GII.2 | GII.P16 | Novel |
| Osceola-0871-2017 | MK764017 | GII.2 | GII.P16 | Novel |
| Lake-0942-2017 | MK775030 | GII.2 | GII.P16 | Novel |
| Butler-0946-2017 | MK775031 | GII.13 | GII.P16 | Extant B |
| Pinellas-1211-2017 | MK762631 | GII.2 | GII.P16 | Novel |
| Nassau-1362-2016 | MK764042 | GII.2 | GII.P16 | Novel |
| Nassau-1363-2016 | MK764043 | GII.2 | GII.P16 | Novel |
| CS-3373-2018 | MK773571 | GII.2 | GII.P16 | Novel |
| Chittenden-0716-2017 | MK762634 | GII.2 | GII.P16 | Novel |

* Near complete sequences were missing no more than 50 nucleotides at the 5’ or 3’ ends of the coding regions of RdRp or VP1. # All GII.4 viruses belong to the GII.4 Sydney variant.NA: Not applicable.
