# Supplementary material for: Emerging Novel GII.P16 Noroviruses Associated with Multiple Capsid Genotypes
Source: Viruses. 2019 Jun 8;11(6):535. doi: 10.3390/v11060535 (PMC6631344; doi:10.3390/v11060535)
Supplement: Supplementary file 1 [file viruses-11-00535-s001.zip › Supplemental Table 2_Submission_updated.docx]

**Supplemental Table 2**. GenBank accession numbers, norovirus dual-typing information, and GII.P16 subtypes for sequences with near complete or complete VP1 (C-type) and RdRp (P-type) coding regions downloaded from GenBank*

| **GenBank accession** | **C-type** # | **P-type** | **GII.P16 subtype** |
| --- | --- | --- | --- |
| AB972466 | GII.4 | GII.Pe | NA |
| AB972467 | GII.4 | GII.Pe | NA |
| AB972468 | GII.4 | GII.Pe | NA |
| AB972469 | GII.4 | GII.Pe | NA |
| AB972470 | GII.4 | GII.Pe | NA |
| AB972472 | GII.4 | GII.Pe | NA |
| AB972473 | GII.4 | GII.Pe | NA |
| AB972475 | GII.4 | GII.Pe | NA |
| AB972477 | GII.4 | GII.Pe | NA |
| AB972478 | GII.4 | GII.Pe | NA |
| AB972479 | GII.4 | GII.Pe | NA |
| AB972480 | GII.4 | GII.Pe | NA |
| AB972481 | GII.4 | GII.Pe | NA |
| AB972482 | GII.4 | GII.Pe | NA |
| AB972483 | GII.4 | GII.Pe | NA |
| AB972484 | GII.4 | GII.Pe | NA |
| AB972485 | GII.4 | GII.Pe | NA |
| AB972487 | GII.4 | GII.Pe | NA |
| AB972488 | GII.4 | GII.Pe | NA |
| AB972489 | GII.4 | GII.Pe | NA |
| AB972490 | GII.4 | GII.Pe | NA |
| AB972491 | GII.4 | GII.Pe | NA |
| AB972492 | GII.4 | GII.Pe | NA |
| AB972493 | GII.4 | GII.Pe | NA |
| AB972494 | GII.4 | GII.Pe | NA |
| AB972495 | GII.4 | GII.Pe | NA |
| AB972496 | GII.4 | GII.Pe | NA |
| AB972497 | GII.4 | GII.Pe | NA |
| AB972498 | GII.4 | GII.Pe | NA |
| AB972499 | GII.4 | GII.Pe | NA |
| AB972502 | GII.4 | GII.Pe | NA |
| AB972503 | GII.4 | GII.Pe | NA |
| AB972504 | GII.4 | GII.Pe | NA |
| AB972505 | GII.4 | GII.Pe | NA |
| DQ456824 | GII.2 | GII.P2 | NA |
| JX459907 | GII.4 | GII.Pe | NA |
| JX459908 | GII.4 | GII.Pe | NA |
| KC175323 | GII.4 | GII.Pe | NA |
| KC517361 | GII.4 | GII.Pe | NA |
| KC517362 | GII.4 | GII.Pe | NA |
| KC517364 | GII.4 | GII.Pe | NA |
| KC517376 | GII.4 | GII.Pe | NA |
| KC517377 | GII.4 | GII.Pe | NA |
| KC517378 | GII.4 | GII.Pe | NA |
| KC631827 | GII.4 | GII.Pe | NA |
| KC962462 | GII.4 | GII.P4 | NA |
| KF145148 | GII.4 | GII.Pe | NA |
| KF306214 | GII.4 | GII.Pe | NA |
| KF509946 | GII.4 | GII.Pe | NA |
| KF509947 | GII.4 | GII.P4 | NA |
| KF895841 | GII.3 | GII.P16 | Extant B |
| KF944110 | GII.3 | GII.P16 | Extant B |
| KF944111 | GII.3 | GII.P16 | Extant B |
| KJ145322 | GII.13 | GII.P16 | Extant B |
| KJ196279 | GII.4 | GII.Pe | NA |
| KJ196280 | GII.4 | GII.Pe | NA |
| KJ196281 | GII.4 | GII.Pe | NA |
| KJ196283 | GII.4 | GII.Pe | NA |
| KJ196286 | GII.17 | GII.P16 | Extant A |
| KJ196293 | GII.4 | GII.Pe | NA |
| KJ196296 | GII.4 | GII.Pe | NA |
| KJ407074 | GII.2 | GII.P16 | Extant B |
| KJ451059 | GII.4 | GII.Pe | NA |
| KJ451060 | GII.4 | GII.Pe | NA |
| KJ533132 | GII.4 | GII.Pe | NA |
| KJ533133 | GII.4 | GII.Pe | NA |
| KJ533134 | GII.4 | GII.Pe | NA |
| KJ649705 | GII.4 | GII.Pe | NA |
| KJ685402 | GII.4 | GII.P4 | NA |
| KJ685406 | GII.4 | GII.Pe | NA |
| KJ685411 | GII.4 | GII.P4 | NA |
| KJ685412 | GII.4 | GII.Pe | NA |
| KJ955492 | GII.4 | GII.Pe | NA |
| KJ955493 | GII.4 | GII.Pe | NA |
| KM036380 | GII.13 | GII.P16 | Extant B |
| KM245075 | GII.4 | GII.Pe | NA |
| KM258128 | GII.4 | GII.Pe | NA |
| KM258129 | GII.4 | GII.Pe | NA |
| KM272334 | GII.4 | GII.Pe | NA |
| KP784694 | GII.4 | GII.P4 | NA |
| KP784696 | GII.4 | GII.Pe | NA |
| KT202793 | GII.4 | GII.Pe | NA |
| KT202794 | GII.4 | GII.Pe | NA |
| KT202795 | GII.4 | GII.Pe | NA |
| KT202796 | GII.4 | GII.Pe | NA |
| KT202797 | GII.4 | GII.Pe | NA |
| KT202798 | GII.4 | GII.Pe | NA |
| KT589391 | GII.17 | GII.Pe | NA |
| KT779557 | GII.3 | GII.P16 | Extant B |
| KU678201 | GII.4 | GII.Pe | NA |
| KU678202 | GII.4 | GII.Pe | NA |
| KU678203 | GII.4 | GII.Pe | NA |
| KU678204 | GII.4 | GII.Pe | NA |
| KU678205 | GII.4 | GII.Pe | NA |
| KX158279 | GII.4 | GII.Pe | NA |
| KX158280 | GII.4 | GII.Pe | NA |
| KX158283 | GII.4 | GII.Pe | NA |
| KX158285 | GII.4 | GII.Pe | NA |
| KX586330 | GII.4 | GII.Pe | NA |
| KX657722 | GII.4 | GII.Pe | NA |
| KX657723 | GII.4 | GII.Pe | NA |
| KX657724 | GII.4 | GII.Pe | NA |
| KX657725 | GII.4 | GII.Pe | NA |
| KX657726 | GII.4 | GII.Pe | NA |
| KX657727 | GII.4 | GII.Pe | NA |
| KX657728 | GII.4 | GII.Pe | NA |
| KX657729 | GII.4 | GII.Pe | NA |
| KX657730 | GII.4 | GII.Pe | NA |
| KX657731 | GII.4 | GII.Pe | NA |
| KX657732 | GII.4 | GII.Pe | NA |
| KX657733 | GII.4 | GII.Pe | NA |
| KX657735 | GII.4 | GII.Pe | NA |
| KX657736 | GII.4 | GII.Pe | NA |
| KX657737 | GII.4 | GII.Pe | NA |
| KX907727 | GII.4 | GII.P16 | Novel |
| KY421039 | GII.4 | GII.Pe | NA |
| KY421121 | GII.2 | GII.P16 | Novel |
| KY421122 | GII.2 | GII.P16 | Novel |
| KY424328 | GII.4 | GII.Pe | NA |
| KY424329 | GII.4 | GII.Pe | NA |
| KY457726 | GII.2 | GII.P16 | Novel |
| KY457727 | GII.2 | GII.P16 | Novel |
| KY457729 | GII.2 | GII.P16 | Novel |
| KY457734 | GII.2 | GII.P16 | Novel |
| KY486271 | GII.4 | GII.Pe | NA |
| KY488570 | GII.4 | GII.Pe | NA |
| KY488572 | GII.4 | GII.Pe | NA |
| KY488573 | GII.4 | GII.Pe | NA |
| KY488574 | GII.4 | GII.Pe | NA |
| KY488575 | GII.4 | GII.Pe | NA |
| KY496327 | GII.4 | GII.Pe | NA |
| KY771081 | GII.2 | GII.P16 | Novel |
| KY865306 | GII.2 | GII.P16 | Novel |
| KY865307 | GII.2 | GII.P16 | Novel |
| KY887597 | GII.3 | GII.P16 | Novel |
| KY887598 | GII.3 | GII.P16 | Novel |
| KY887599 | GII.4 | GII.P16 | Novel |
| KY887600 | GII.4 | GII.P16 | Novel |
| KY887601 | GII.4 | GII.P16 | Novel |
| KY887602 | GII.4 | GII.P16 | Novel |
| KY887603 | GII.4 | GII.P16 | Novel |
| KY887604 | GII.4 | GII.P16 | Novel |
| KY887605 | GII.4 | GII.P16 | Novel |
| KY887606 | GII.3 | GII.P16 | Novel |
| KY905333 | GII.4 | GII.Pe | NA |
| KY905335 | GII.4 | GII.P16 | Novel |
| KY905336 | GII.2 | GII.P16 | Novel |
| KY905337 | GII.2 | GII.P16 | Novel |
| KY905338 | GII.2 | GII.P16 | Novel |
| KY947546 | GII.4 | GII.P4 | NA |
| KY947547 | GII.4 | GII.P4 | NA |
| KY947548 | GII.13 | GII.P16 | Extant B |
| KY947549 | GII.4 | GII.P16 | Novel |
| KY947550 | GII.4 | GII.P16 | Novel |
| LC066046 | GII.4 | GII.Pe | NA |
| LC133344 | GII.4 | GII.Pe | NA |
| LC145786 | GII.2 | GII.P16 | Extant A |
| LC145787 | GII.2 | GII.P16 | Extant B |
| LC145788 | GII.2 | GII.P16 | Extant A |
| LC145789 | GII.2 | GII.P16 | Extant A |
| LC145790 | GII.2 | GII.P16 | Extant A |
| LC145791 | GII.2 | GII.P16 | Extant A |
| LC145792 | GII.2 | GII.P16 | Extant A |
| LC145793 | GII.2 | GII.P16 | Extant A |
| LC145794 | GII.2 | GII.P16 | Extant A |
| LC145795 | GII.2 | GII.P16 | Extant A |
| LC145796 | GII.2 | GII.P16 | Extant A |
| LC145797 | GII.2 | GII.P16 | Extant A |
| LC145798 | GII.2 | GII.P16 | Extant A |
| LC145799 | GII.2 | GII.P16 | Extant A |
| LC145800 | GII.2 | GII.P16 | Extant A |
| LC145801 | GII.2 | GII.P16 | Extant A |
| LC145802 | GII.2 | GII.P16 | Extant A |
| LC145803 | GII.2 | GII.P16 | Extant A |
| LC145804 | GII.2 | GII.P16 | Extant A |
| LC145805 | GII.2 | GII.P16 | Extant A |
| LC145806 | GII.2 | GII.P16 | Extant A |
| LC145807 | GII.2 | GII.P16 | Extant A |
| LC145808 | GII.2 | GII.P16 | Extant A |
| LC175468 | GII.4 | GII.P16 | Novel |
| LC209431 | GII.2 | GII.P16 | Extant A |
| LC209432 | GII.2 | GII.P16 | Extant A |
| LC209433 | GII.2 | GII.P16 | Extant A |
| LC209434 | GII.2 | GII.P16 | Extant A |
| LC209436 | GII.2 | GII.P2 | NA |
| LC209437 | GII.2 | GII.P2 | NA |
| LC209438 | GII.2 | GII.P2 | NA |
| LC209440 | GII.2 | GII.P2 | NA |
| LC209441 | GII.2 | GII.P16 | Extant A |
| LC209444 | GII.2 | GII.P16 | Extant A |
| LC209445 | GII.2 | GII.P16 | Extant A |
| LC209446 | GII.2 | GII.P16 | Extant B |
| LC209447 | GII.2 | GII.P16 | Extant B |
| LC209448 | GII.2 | GII.P16 | Extant B |
| LC209449 | GII.2 | GII.P16 | Extant B |
| LC209450 | GII.2 | GII.P16 | Extant A |
| LC209451 | GII.2 | GII.P16 | Extant B |
| LC209452 | GII.2 | GII.P16 | Extant B |
| LC209453 | GII.2 | GII.P16 | Extant B |
| LC209454 | GII.2 | GII.P16 | Extant A |
| LC209455 | GII.2 | GII.P16 | Extant A |
| LC209456 | GII.2 | GII.P16 | Extant A |
| LC209457 | GII.2 | GII.P2 | NA |
| LC209458 | GII.2 | GII.P16 | Extant A |
| LC209459 | GII.2 | GII.P16 | Extant B |
| LC209460 | GII.2 | GII.P16 | Extant B |
| LC209461 | GII.2 | GII.P16 | Extant A |
| LC209462 | GII.2 | GII.P2 | NA |
| LC209463 | GII.2 | GII.P2 | NA |
| LC209464 | GII.2 | GII.P2 | NA |
| LC209465 | GII.2 | GII.P2 | NA |
| LC209466 | GII.2 | GII.P16 | Extant A |
| LC209467 | GII.2 | GII.P16 | Extant B |
| LC209468 | GII.2 | GII.P16 | Extant B |
| LC209469 | GII.2 | GII.P2 | NA |
| LC209470 | GII.2 | GII.P16 | Extant A |
| LC209471 | GII.2 | GII.P16 | Extant B |
| LC209472 | GII.2 | GII.P2 | NA |
| LC209473 | GII.2 | GII.P2 | NA |
| LC209474 | GII.2 | GII.P2 | NA |
| LC209475 | GII.2 | GII.P16 | Extant A |
| LC209476 | GII.2 | GII.P16 | Extant A |
| LC209477 | GII.2 | GII.P16 | Extant A |
| LC209478 | GII.2 | GII.P16 | Extant B |
| LC209479 | GII.2 | GII.P16 | Extant B |
| LC209480 | GII.2 | GII.P16 | Extant B |
| LC209481 | GII.2 | GII.P16 | Extant B |
| LC213885 | GII.2 | GII.P16 | Extant A |
| LC213886 | GII.2 | GII.P16 | Novel |
| LC213887 | GII.2 | GII.P16 | Novel |
| LC213888 | GII.2 | GII.P16 | Novel |
| LC213889 | GII.2 | GII.P16 | Novel |
| LC213890 | GII.2 | GII.P16 | Novel |
| LC213891 | GII.2 | GII.P16 | Novel |
| LC213892 | GII.2 | GII.P16 | Novel |
| LC213893 | GII.2 | GII.P16 | Novel |
| LC213894 | GII.2 | GII.P16 | Novel |
| LC213895 | GII.2 | GII.P16 | Novel |
| LC213896 | GII.2 | GII.P16 | Novel |
| LC213897 | GII.2 | GII.P16 | Novel |
| LC213898 | GII.2 | GII.P16 | Novel |
| LC213899 | GII.2 | GII.P16 | Novel |
| LC213900 | GII.2 | GII.P16 | Novel |
| LC213901 | GII.2 | GII.P16 | Novel |
| LC215413 | GII.2 | GII.P16 | Novel |
| LC215414 | GII.2 | GII.P16 | Novel |
| LC215415 | GII.2 | GII.P16 | Novel |
| LC228948 | GII.2 | GII.P16 | Extant A |
| LC279234 | GII.2 | GII.P16 | Novel |
| LC279235 | GII.2 | GII.P16 | Novel |
| LC279236 | GII.2 | GII.P16 | Novel |
| LC279237 | GII.2 | GII.P16 | Novel |
| LC279238 | GII.2 | GII.P16 | Novel |
| LC279239 | GII.2 | GII.P16 | Novel |
| LC279240 | GII.2 | GII.P16 | Novel |
| LC279241 | GII.2 | GII.P16 | Novel |
| LC279242 | GII.2 | GII.P16 | Novel |
| LC279243 | GII.2 | GII.P16 | Novel |
| LC333898 | GII.17 | GII.Pe | NA |
| LC349981 | GII.2 | GII.P16 | Novel |
| LC349982 | GII.2 | GII.P16 | Novel |
| LC349983 | GII.2 | GII.P16 | Novel |
| LC349984 | GII.2 | GII.P16 | Novel |
| LC349985 | GII.2 | GII.P16 | Novel |
| LN854566 | GII.4 | GII.P4 | NA |
| LN854567 | GII.4 | GII.Pe | NA |
| MF167650 | GII.2 | GII.P16 | Novel |
| MF167651 | GII.2 | GII.P16 | Novel |
| MF167652 | GII.2 | GII.P16 | Novel |
| MF621948 | GII.2 | GII.P16 | Extant A |
| MF621949 | GII.2 | GII.P16 | Extant A |
| MF621950 | GII.2 | GII.P16 | Extant A |
| MF621951 | GII.2 | GII.P16 | Extant A |
| MF621952 | GII.2 | GII.P16 | Extant A |
| MF621953 | GII.2 | GII.P16 | Extant A |
| MF621954 | GII.2 | GII.P16 | Extant A |
| MF621955 | GII.2 | GII.P16 | Extant A |
| MF621956 | GII.2 | GII.P16 | Extant A |
| MF802551 | GII.2 | GII.P16 | Novel |
| MG002630 | GII.4 | GII.P16 | Novel |
| MG002631 | GII.4 | GII.P16 | Novel |
| MG002632 | GII.4 | GII.P4 | NA |
| MG002633 | GII.4 | GII.P16 | Novel |
| MG002634 | GII.4 | GII.P4 | NA |
| MG214988 | GII.4 | GII.Pe | NA |
| MG557655 | GII.10 | GII.Pe | NA |
| MG572182 | GII.1 | GII.P16 | Novel |
| MG745985 | GII.2 | GII.P16 | Novel |
| MG745986 | GII.2 | GII.P16 | Novel |
| MG745987 | GII.2 | GII.P16 | Novel |
| MG745988 | GII.2 | GII.P16 | Novel |
| MG745989 | GII.2 | GII.P16 | Novel |
| MG745990 | GII.2 | GII.P16 | Novel |
| MG745991 | GII.2 | GII.P16 | Novel |
| MG745992 | GII.2 | GII.P16 | Novel |
| MG745993 | GII.2 | GII.P16 | Novel |
| MG745994 | GII.2 | GII.P16 | Novel |
| MG745995 | GII.2 | GII.P16 | Novel |
| MG745996 | GII.2 | GII.P16 | Novel |
| MG745997 | GII.2 | GII.P16 | Novel |
| MG745998 | GII.2 | GII.P16 | Novel |
| MG745999 | GII.2 | GII.P16 | Novel |
| MG746000 | GII.2 | GII.P16 | Novel |
| MG746001 | GII.2 | GII.P16 | Novel |
| MG746002 | GII.2 | GII.P16 | Novel |
| MG746003 | GII.2 | GII.P16 | Novel |
| MG746004 | GII.2 | GII.P16 | Novel |
| MG746005 | GII.2 | GII.P16 | Novel |
| MG746006 | GII.2 | GII.P16 | Novel |
| MG746007 | GII.2 | GII.P16 | Novel |
| MG746008 | GII.2 | GII.P16 | Novel |
| MG746009 | GII.2 | GII.P16 | Novel |
| MG746010 | GII.2 | GII.P16 | Novel |
| MG746011 | GII.2 | GII.P16 | Novel |
| MG746012 | GII.2 | GII.P16 | Novel |
| MG746013 | GII.2 | GII.P16 | Novel |
| MG746014 | GII.2 | GII.P16 | Novel |
| MG746015 | GII.2 | GII.P16 | Novel |
| MG746016 | GII.2 | GII.P16 | Novel |
| MG746017 | GII.2 | GII.P16 | Novel |
| MG746018 | GII.2 | GII.P16 | Novel |
| MG746019 | GII.2 | GII.P16 | Novel |
| MG746020 | GII.2 | GII.P16 | Novel |
| MG746021 | GII.2 | GII.P16 | Novel |
| MG746022 | GII.2 | GII.P16 | Novel |
| MG746023 | GII.2 | GII.P16 | Novel |
| MG746024 | GII.2 | GII.P16 | Novel |
| MG746025 | GII.2 | GII.P16 | Novel |
| MG746026 | GII.2 | GII.P16 | Novel |
| MG746027 | GII.2 | GII.P16 | Novel |
| MG746028 | GII.2 | GII.P16 | Novel |
| MG746029 | GII.2 | GII.P16 | Novel |
| MG746030 | GII.2 | GII.P16 | Novel |
| MG746031 | GII.2 | GII.P16 | Novel |
| MG746032 | GII.2 | GII.P16 | Novel |
| MG746033 | GII.2 | GII.P16 | Novel |
| MG746034 | GII.2 | GII.P16 | Novel |
| MG746035 | GII.2 | GII.P16 | Novel |
| MG746036 | GII.2 | GII.P16 | Novel |
| MG746037 | GII.2 | GII.P16 | Novel |
| MG746038 | GII.2 | GII.P16 | Novel |
| MG746039 | GII.2 | GII.P16 | Novel |
| MG746040 | GII.2 | GII.P16 | Novel |
| MG746041 | GII.2 | GII.P16 | Novel |
| MG746042 | GII.2 | GII.P16 | Novel |
| MG746043 | GII.2 | GII.P16 | Novel |
| MG746044 | GII.2 | GII.P16 | Novel |
| MG746045 | GII.2 | GII.P16 | Novel |
| MG786781 | GII.4 | GII.Pe | NA |
| MH188080 | GII.4 | GII.P4 | NA |
| MH218591 | GII.17 | GII.P16 | Extant A |
| MH218605 | GII.4 | GII.Pe | NA |
| MH218606 | GII.4 | GII.P4 | NA |
| MH218607 | GII.4 | GII.P4 | NA |
| MH218608 | GII.4 | GII.Pe | NA |
| MH218609 | GII.4 | GII.P4 | NA |
| MH218610 | GII.4 | GII.P4 | NA |
| MH218611 | GII.4 | GII.P4 | NA |
| MH218612 | GII.4 | GII.P4 | NA |
| MH218613 | GII.4 | GII.P4 | NA |
| MH218614 | GII.4 | GII.Pe | NA |
| MH218615 | GII.4 | GII.Pe | NA |
| MH218616 | GII.4 | GII.Pe | NA |
| MH218617 | GII.4 | GII.P4 | NA |
| MH218619 | GII.4 | GII.P4 | NA |
| MH218620 | GII.4 | GII.Pe | NA |
| MH218621 | GII.4 | GII.Pe | NA |
| MH218622 | GII.4 | GII.Pe | NA |
| MH218623 | GII.4 | GII.Pe | NA |
| MH218624 | GII.4 | GII.Pe | NA |
| MH218625 | GII.4 | GII.Pe | NA |
| MH218626 | GII.4 | GII.Pe | NA |
| MH218627 | GII.4 | GII.Pe | NA |
| MH218628 | GII.4 | GII.Pe | NA |
| MH218629 | GII.4 | GII.Pe | NA |
| MH218631 | GII.4 | GII.Pe | NA |
| MH218632 | GII.4 | GII.Pe | NA |
| MH218633 | GII.4 | GII.P4 | NA |
| MH218634 | GII.4 | GII.Pe | NA |
| MH218635 | GII.4 | GII.Pe | NA |
| MH218636 | GII.4 | GII.P4 | NA |
| MH218637 | GII.4 | GII.Pe | NA |
| MH218638 | GII.4 | GII.Pe | NA |
| MH218652 | GII.4 | GII.P4 | NA |
| MH218655 | GII.2 | GII.P2 | NA |
| MH218662 | GII.4 | GII.Pe | NA |
| MH218663 | GII.4 | GII.Pe | NA |
| MH218665 | GII.4 | GII.P4 | NA |
| MH218670 | GII.4 | GII.P4 | NA |
| MH218674 | GII.4 | GII.Pe | NA |
| MH218684 | GII.4 | GII.P16 | Novel |
| MH218685 | GII.4 | GII.P16 | Novel |
| MH218691 | GII.4 | GII.P4 | NA |
| MH218694 | GII.4 | GII.Pe | NA |
| MH218695 | GII.4 | GII.P4 | NA |
| MH218698 | GII.4 | GII.P4 | NA |
| MH218699 | GII.4 | GII.Pe | NA |
| MH218700 | GII.4 | GII.Pe | NA |
| MH218701 | GII.4 | GII.Pe | NA |
| MH218702 | GII.4 | GII.Pe | NA |
| MH218703 | GII.4 | GII.P4 | NA |
| MH218704 | GII.4 | GII.P4 | NA |
| MH218705 | GII.4 | GII.Pe | NA |
| MH218706 | GII.4 | GII.Pe | NA |
| MH218707 | GII.4 | GII.Pe | NA |
| MH218708 | GII.4 | GII.Pe | NA |
| MH218709 | GII.4 | GII.Pe | NA |
| MH218710 | GII.4 | GII.P4 | NA |
| MH218711 | GII.4 | GII.Pe | NA |
| MH218713 | GII.4 | GII.P4 | NA |
| MH218714 | GII.4 | GII.P4 | NA |
| MH218715 | GII.4 | GII.Pe | NA |
| MH218716 | GII.4 | GII.P4 | NA |
| MH218718 | GII.4 | GII.P4 | NA |
| MH218734 | GII.2 | GII.P2 | NA |
| MH218735 | GII.2 | GII.P2 | NA |
| MH218736 | GII.2 | GII.P2 | NA |
| MH218737 | GII.2 | GII.P2 | NA |
| MH260479 | GII.4 | GII.P16 | Novel |
| MH260480 | GII.4 | GII.P16 | Novel |
| MH260483 | GII.4 | GII.P16 | Novel |
| MH260484 | GII.4 | GII.P16 | Novel |
| MH260485 | GII.4 | GII.P16 | Novel |
| MH260486 | GII.4 | GII.P16 | Novel |
| MH260488 | GII.4 | GII.P16 | Novel |
| MH260489 | GII.4 | GII.P16 | Novel |
| MH260490 | GII.4 | GII.P16 | Novel |
| MH260491 | GII.4 | GII.P16 | Novel |
| MH260492 | GII.4 | GII.P16 | Novel |
| MH260493 | GII.2 | GII.P16 | Novel |
| MH260495 | GII.4 | GII.P16 | Novel |
| MH260496 | GII.4 | GII.P16 | Novel |
| MH260497 | GII.4 | GII.P16 | Novel |
| MH260498 | GII.4 | GII.P16 | Novel |
| MH260499 | GII.4 | GII.P16 | Novel |
| MH260500 | GII.2 | GII.P16 | Novel |
| MH260501 | GII.2 | GII.P16 | Novel |
| MH260502 | GII.4 | GII.P16 | Novel |
| MH260503 | GII.4 | GII.P16 | Novel |
| MH260504 | GII.4 | GII.P16 | Novel |
| MH260505 | GII.4 | GII.P16 | Novel |
| MH260506 | GII.4 | GII.P16 | Novel |
| MH260508 | GII.4 | GII.P16 | Novel |
| MH260509 | GII.2 | GII.P16 | Novel |
| MH279823 | GII.4 | GII.P16 | Novel |
| MH279825 | GII.4 | GII.P16 | Novel |
| MH321825 | GII.2 | GII.P16 | Novel |
| MH608287 | GII.13 | GII.P16 | Extant B |

* Near complete sequences were missing no more than 50 nucleotides at the 5’ or 3’ ends of the coding regions of RdRp and VP1. # All GII.4 viruses belong to the GII.4 Sydney variant. NA: Not applicable.
